# Clinical value of ultrasound-guided full-needle path anesthesia in transperineal prostate biopsy: An observational study

**DOI:** 10.1097/MD.0000000000039008

**Published:** 2024-07-19

**Authors:** DianYuan Lu, JunYu Zhou, JianRong Cai, Lan Liu, Ye Ni

**Affiliations:** a Department of Ultrasound, Chongming Hospital Affiliated to Shanghai University of Medicine and Health Sciences, Shanghai, China.

**Keywords:** biopsy, full-needle path, pain, prostate cancer, transperineal, visual analog scale (VAS)

## Abstract

**Background:**

The pain sensation in a transperineal prostate biopsy was obvious. This study explored the clinical value of ultrasound-guided full-needle path anesthesia in transperineal prostate biopsy.

**Methods:**

Two hundred patients who underwent ultrasound-guided transperineal prostate biopsy at our department were randomly divided into 2 groups. The control group received routine local infiltration anesthesia, and the experimental group received ultrasound-guided full-needle path anesthesia. Immediately after biopsy, visual analog scoring was used to evaluate pain during the biopsy process. Seven days postbiopsy, telephone follow-up revealed symptoms, such as hematuria and discomfort during urination. The measured data were expressed as x ± s. The 2 groups were compared using the *t* test, and the differences were statistically significant (*P* < .05).

**Results:**

There were no significant differences in age, prostate-specific antigen (PSA) level, or prostate volume between the 2 groups, and all patients underwent prostate biopsy. The pain score of visual analog score was (2.55 ± 0.88), urination discomfort was (1.86 ± 0.67) days and hematuria time was (2.87 ± 0.91) days in the experimental group after biopsy. In the control group, the pain score of visual analog scale was (4.32 ± 0.94), the urination discomfort was (2.3 ± 0.77) days, and the hematuria time was (2.85 ± 0.83) days. Pain scores and urination discomfort were compared between the 2 groups (*P* < .01). Pain and urination discomfort associated with prostate biopsy in the experimental group were significantly lower than those in the control group.

**Conclusion:**

Ultrasound-guided full needle path anesthesia can alleviate pain sensation in patients undergoing transperineal prostate biopsy and has high clinical value.

## 1. Introduction

Prostate cancer (PCa) is one of the most common malignant tumors in elderly men, and its incidence rate ranks second among all malignant tumors in men worldwide.^[1]^ Siegel et al^[2]^ showed that new cases of PCa accounted for approximately 27% of all male tumor patients, and the case fatality rate accounted for 9% of male tumor deaths, second only to lung tumor deaths. In recent years, with the formation of an aging society in China, improvement in economic living standards, and development of medical technology, the incidence rate of PCa has also shown a significant upward trend.^[3]^ Transrectal ultrasound-guided prostate biopsy is the gold standard for clinical diagnosis of PCa.^[4]^ Currently, there are 2 commonly used approaches in clinical practice: transrectal and transperineal. In recent years, research reports^[5–8]^ that the transperineal approach has received increasing attention and recognition because of its higher safety in tumor detection and puncture than the transrectal approach. However, the pain sensation during the puncture process through the transperineal approach is higher than that through the transrectal approaches,^[9]^ and there is currently no standard local anesthesia method for this surgery. This study aimed to identify a more suitable anesthesia method by comparing the effects of ultrasound-guided full-needle path anesthesia with those of conventional local infiltration anesthesia.

## 2. Materials and methods

All patients in this retrospective study signed informed consent forms before biopsy.

### 2.1. Patients

Two hundred patients who underwent ultrasound-guided transperineal prostate biopsy at our department between January 2020 and December 2021 were randomly divided into 2 groups. All patients signed informed consent forms before biopsy. The Ethics Committee of Chongming Hospital Affiliated to Shanghai University School of Medicine and Science has approved this study. The experimental group consisted of 100 patients with an average age of 79.36 ± 5.17. There were 100 cases in the control group, with an average age of 78.07 ± 4.48. The patient had not received any clinical treatment for prostate disease before seeking medical attention. The exclusion criteria were a history of prostate biopsy and diseases that can affect the patient’s pain during puncture, such as combined hemorrhoids, anal stenosis, colorectal cancer, inflammatory bowel disease, urinary stones, and other diseases.

### 2.2. Biopsy equipments

Ultrasound-guided transperineal prostate biopsies were performed using a Mylab™ Twice (EsaoteSpA, Genoa, Italy) scanner with a TRT33 biplane probe. The biopsy needle was a TSK 18 gauge × 160 mm equipped with a spring-loaded biopsy gun (BARD Maxcore, Covington, GA).

### 2.3. Anesthesia methods

**Figure 1.**
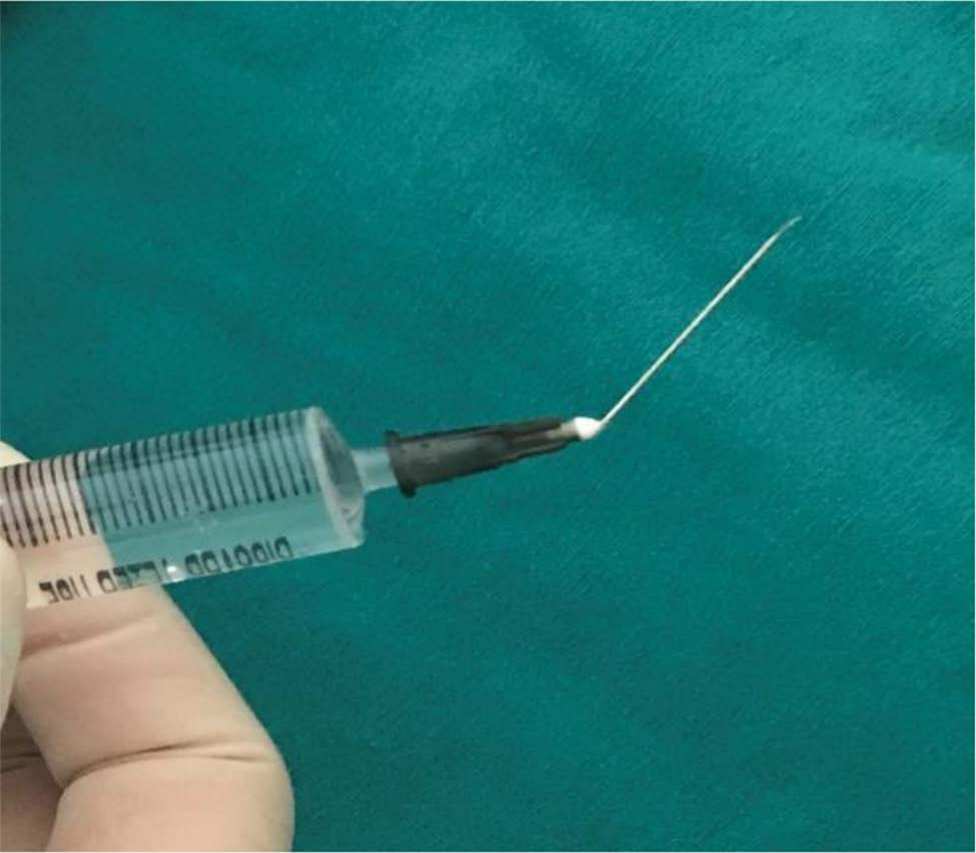
The injection needle is bent 45° toward the inclined direction of the needle tip. When the needle tip enters the subcutaneous tissue, it is basically parallel to the epidermis, which is more conducive to increasing the area of subcutaneous tissue anesthesia.

**Figure 2.**
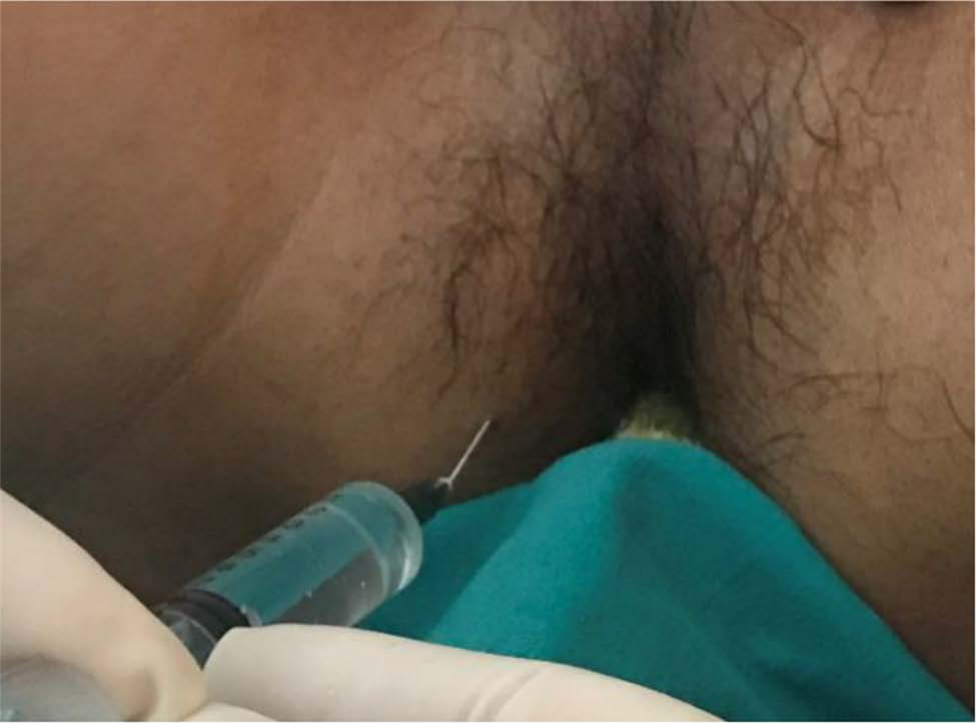
Local anesthetic is injected into subcutaneous tissue 2 cm from the front edge of anus. Because the needle is parallel to the epidermis, and when injecting local anesthetic drugs, the needle is withdrawn while pushing the drug. So only a small amount of local anesthetic needs to be injected to achieve good local anesthetic effects.

**Figure 3.**
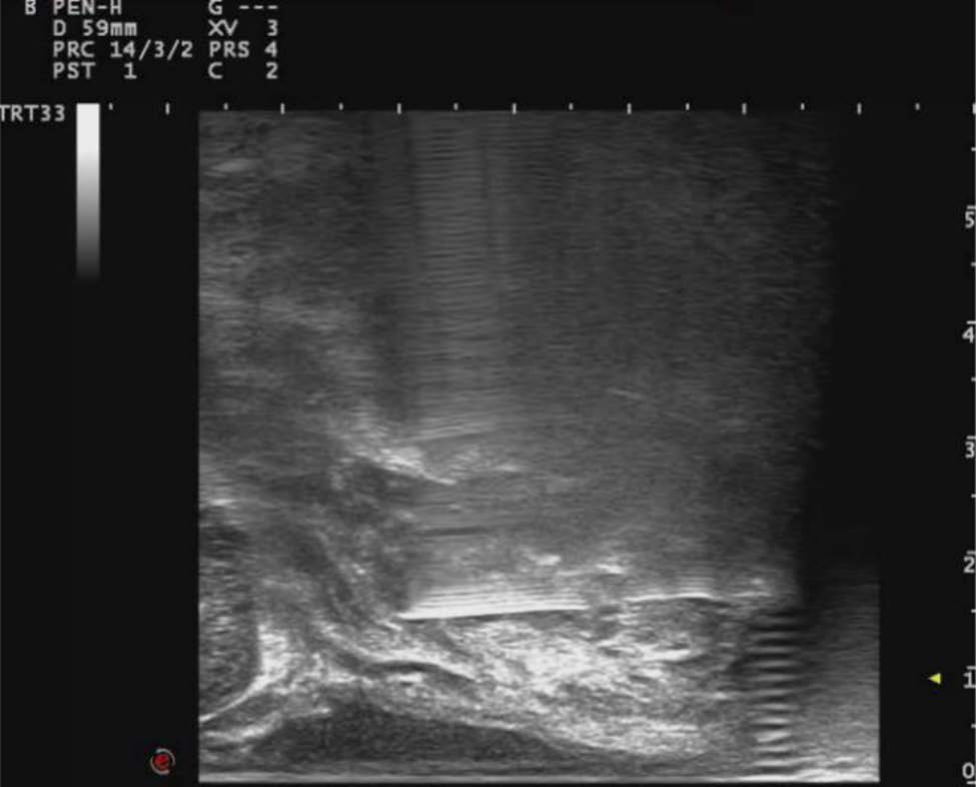
Ultrasound-guided anesthesia for injection of local anesthetics into the puborectal muscle. Under ultrasound guidance, we can evenly inject local anesthetic drugs into the anterior and posterior capsule of the puborectal muscle, and effectively avoid the larger venous plexus in the pelvic floor, reducing the probability of side effects of local anesthetic drugs.

**Figure 4.**
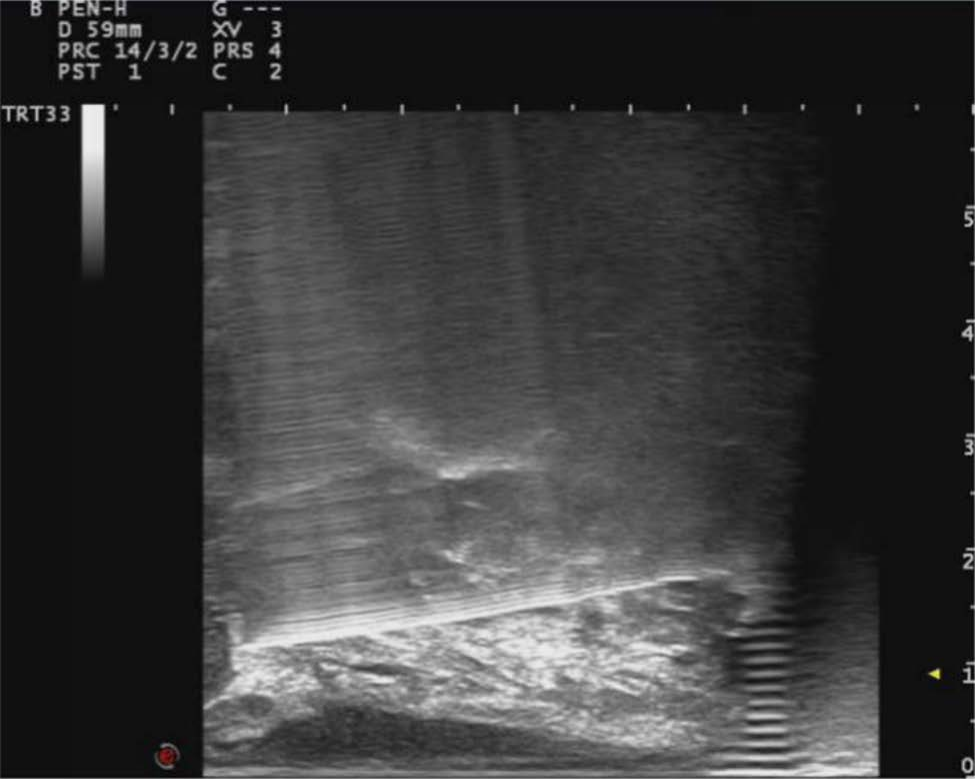
Ultrasound-guided anesthesia for injection of local anesthetics into the prostate capsule. Under ultrasound guidance, we can accurately inject local anesthetic drugs into the prostate capsule, effectively carry out prostate nerve block, and reduce patient pain.

Both groups underwent transperineal prostate biopsy. The patient was placed in a flat lithotomy position. The control group was anesthetized with conventional local infiltration anesthesia, that is, a 10 mL syringe was used to extract lidocaine hydrochloride (5 mL: 0.1 g), 5 mL local anesthetic was injected into the subcutaneous and subcutaneous tissue on both sides of the anus, for a total of 10 mL, and biopsy was performed 5 minutes later. The anesthesia method in the experimental group was ultrasound-guided full-needle path anesthesia, that is, 8 mL of lidocaine hydrochloride (5 mL: 0.1 g) was extracted with a 10 mL syringe, and the injection needle was bent 45° toward the inclined plane of the needle tip (Fig. 1). First, 1 mL local anesthetics were injected into the subcutaneous tissue 2 cm from the front edge of the anus on both sides of the anus (Fig. 2), and then the injection needle was replaced with a disposable anesthesia needle (model and specification: AN-S 0.7 × 90), Under the guidance of transrectal ultrasound, 3 mL of local anesthetics were uniformly injected into the periphery of the puborectal muscle, the prostate capsule, and the needle path on both sides (Figs. 3 and 4). Biopsies were performed after 5 minutes. The puncture mode in both groups was 10+ ×.

### 2.4. Data collection

After the biopsy was completed, both groups of patients immediately used the visual analog scale (VAS) to score pain during the biopsy process. The scoring method divided the degree of pain into 0 to 10 points, with 0 representing no pain, 1 to 3 representing mild pain, 4 to 6 representing moderate pain, and 7 to 10 representing severe pain. Seven days after the biopsy, follow-up calls were made to address symptoms, such as hematuria and discomfort during urination.

### 2.5. Statistical analysis

All statistical analyses were performed using SPSS software (version 17.0; SPSS, Chicago, IL). Quantitative data are expressed as mean ± standard deviation. Statistical significance was set at *P* < .05.

## 3. Results

Both groups of patients were able to complete prostate biopsy. Routine data of the 2 groups of patients (Table 1), VAS scores, days of urinary discomfort, and days of hematuria (Table 2).

**Table 1:** Routine data of 2 groups of patients.

	Age (yr)	PSA value (ng/mL)	Prostate volume (mL)
Control group	78.07 ± 4.48	35.86 ± 20.14	36.43 ± 8.82
Experimental group	79.36 ± 5.17*	36.41 ± 17.87*	37.6 ± 9.47*

* Comparison between the 2 groups, *P* > .05.

**Table 2:** Postoperative scores of 2 groups of patients.

	VAS score	Urinary discomfort (d)	Hematuria (d)
Control group	4.32 ± 0.94	2.3 ± 0.77	2.85 ± 0.83
Experimental group	2.55 ± 0.88#	1.86 ± 0.67#	2.87 ± 0.91*

Comparison between 2 groups.

* *P* > .05.

# *P* < .01.

## 4. Discussion

PCa is a common malignant tumor in men. In recent years, the incidence of this disease has been continuously increasing, and it has become the second most common cancer among men worldwide. Therefore, early detection and treatment of PCa are crucial. As the gold standard for the diagnosis of PCa, transrectal ultrasound-guided prostate biopsy is considered the safest and most effective method for diagnosing PCa because of its mature technology and high diagnostic rate.^[10]^ At present, the transrectal and transperineal approaches are 2 commonly used approaches for prostate biopsy, and the transrectal approach has gradually become widely accepted because of its advantages of saving time and facilitating implementation. However, in recent years, a comparative study of the 2 puncture routes has shown that the sensitivity of detecting csPCa is higher (86% vs 73%) when using the transperineal approach.^[11]^ This benefit is especially pronounced in anterior tumors.^[12]^ The transperineal approach can provide more accurate sampling of the anterior prostate gland.^[13]^ Some scholars have pointed out^[6,14–19]^ that the postoperative complications of the transperineal approach are lower than those of the transrectal approach, especially serious complications, such as bloody stools, fever, infection risk, and sepsis. The transperineal approach can accurately observe the puncture route and puncture site of the puncture needle, which is conducive to obtaining material from the anterior and transitional areas.^[20]^ Therefore, the transperineal approach has garnered increasing attention.

Although the transperineal approach has obvious advantages, its puncture path requires passing through the skin, fascia, and various layers of muscle tissue, making it pain sensitive. There are reports^[21]^ that the pain level after surgery using the transperineal approach is significantly higher than that using the transrectal approach. And studies^[22,23]^ have also confirmed that the degree of pain increases with the number of puncture needles. Patients undergoing prostate biopsy are mostly elderly with weak tolerance, and higher levels of pain may affect the accuracy of biopsy and postoperative recovery.^[24]^

Various anesthesia methods have been reported to alleviate pain associated with the transperineal approach, including intraspinal anesthesia, pudendal nerve block, prostate peripheral nerve block, and general anesthesia; however, the analgesic effects of these methods have been reported differently. Previous studies have shown that TPBx performed under local anesthesia is reasonably tolerated by patients,^[25,26]^ and the National Cancer Comprehensive Network of the United States believes that both peripheral prostate nerve block and local anesthesia are effective and safe. A study to anesthetize the branches of the perineal nerve, which involved injection of lidocaine between the deep layer of the superficial fascia and the prostate capsule under transrectal ultrasound guidance, was found to maximize the attenuation of pain associated with transperineal biopsy.^[27]^For patients with a low pain threshold and a large number of puncture needles, general anesthesia should also be considered. At present, the Chinese Society of Urology has not yet defined the anesthesia method used.

Our department has conducted ultrasound-guided transperineal prostate biopsies since 2013, with an average of approximately 300 cases completed annually. In the early stages, our department used only a single anesthesia method, namely conventional local infiltration anesthesia. Because the needle tip length of the syringe was approximately 3 cm, the anesthetic effect on the deep tissues was poor, and the patient’s pain was more obvious. However, for spinal and general anesthesia, owing to the complexity of the operation and high technical requirements, it is not suitable for our department to apply them alone. In a later study, we found that the patient’s pain mainly came from skin breaking during puncture, the puncture needle passing through the puborectal muscle, and the puncture needle entering the prostate capsule during emission. Studies^[28]^ have also suggested that the somatic nerve located at the tip of the prostate is one of the most painful areas during biopsy, and the literature^[29,30]^ has reported that the Te apex prostate never block (PNB) is commonly performed before local transaminal biopsy. Our department innovatively uses ultrasound-guided full-needle path anesthesia based on our own characteristics. First, we bent the injection needle at 45° to the inclined plane of the needle tip for local infiltration anesthesia of the subcutaneous tissue. A bent injection needle can better expand the scope of local anesthesia, make the distribution of local anesthetic drugs more uniform, and reduce pain when the puncture needle breaks the skin. Once again, we chose to use anesthesia needles under real-time ultrasound guidance for local anesthesia of the puncture needle passage. Ultimately, the anesthesia needles arrived at the tip of the prostate. It is a branch of the perineal nerve. Lidocaine (8 mL was used in the experimental group, which was lower than that used in the control group (10 mL)). In the control study of this group, the VAS score of the experimental group was significantly lower than that of the control group, which was similar to the VAS score reported by Stefanova et al^[7]^ but the latter was administered 25 mL of local anesthetic at the time of biopsy, which greatly increased the probability of narcotic adverse reactions. In our early work, we used 20 mL of local anesthetic to reduce the pain of biopsy. At that time, side effects of local anesthetics often occurred, manifested as nausea and vomiting, and significantly reduced patient comfort. In this study, the number of days of postoperative urinary discomfort in the experimental group was significantly lower than that in the control group, indicating that reduction in intraoperative pain is helpful for rapid postoperative recovery. There was no statistically significant difference in hematuria between the 2 groups of patients. Considering that the biopsy time and biopsy method of the 2 groups of patients were not different, there was no difference in hematuria.

This study has certain limitations. First, the number of patients was relatively small, which may have led to a bias. Second, this study was conducted at a single center, and there may be socioeconomic and regional differences. Third, this study only provided a comprehensive VAS score for the biopsy process without subdividing the VAS scores during local anesthesia before and after biopsy.

The results of this controlled study showed that compared to single local anesthesia, ultrasound-guided full-needle path anesthesia can effectively reduce patients’ pain sensation without increasing operational difficulty, providing better anesthesia services for patients undergoing transperineal prostate biopsy, and has high clinical application value.

## Author contributions

**Conceptualization:** Dian-Yuan Lu.

**Data curation:** Dian-Yuan Lu, Jianrong Cai.

**Writing – original draft:** Dian-Yuan Lu, Jun Yu Zhou.

**Investigation:** Jianrong Cai.

**Methodology:** Lan Liu.

**Project administration:** Ye Ni.

**Validation:** Ye Ni.

**Writing – review & editing:** Ye Ni.
